# Electron microscopic analysis of an angiosarcoma of the thyroid from a non-Alpine endemic goiter region: A case report and brief review of the literature

**DOI:** 10.3892/ol.2014.2470

**Published:** 2014-08-21

**Authors:** SERDAR ALTINAY, AYNUR ÖZEN, ESAT NAMAL, PELIN ERTÜRKÜNER

**Affiliations:** 1 Department of Pathology, Thyroid Unit, Bağcılar Training and Education Hospital, Istanbul 34203, Turkey; 2 Department of Nuclear Medicine, Bağcılar Training and Education Hospital, Istanbul 34203, Turkey; 3 Department of Oncology, Bağcılar Training and Education Hospital, Istanbul 34203, Turkey; 4 Department of Histology and Embryology, Medical Faculty of Cerrahpaşa, Istanbul University, Istanbul 34098, Turkey

**Keywords:** thyroid angiosarcoma, non-Alpine, endemic goiter, CD31 antibody

## Abstract

Angiomatoid tumors of the thyroid gland are rare endocrine neoplasms, which exhibit an aggressive behavior. Angiosarcomas of the thyroid are generally reported from the European Alpine region and have a histogenesis that has been under debate for a number of years. The current study presents a rare case of angiosarcoma of the thyroid in a 62-year-old Turkish female. The patient had a 10-year history of goiter and was from the Black Sea region, an endemic goiter region of Turkey. The patient was not taking any medication at the time of admission and swelling had been observed on the right side of the neck throughout the previous few months. Thyroid function tests, which analyzed the levels of thyroid-stimulating hormone, thyroxine and triiodothyronine, were within the normal limits, however, the histopathological findings were consistent with an angiosarcoma of the thyroid. The patient rejected the complementary surgery and chemotherapy options, and is currently disease-free (as per the 15-month follow-up). The current study describes a case of angiosarcoma that was characterized by Weibel-Palade bodies, and light microscopy and immunohistochemical findings, as well as an endothelial origin, which was demonstrated via electron microscopy. To the best of our knowledge, this is the first reported case of angiosarcoma of the thyroid in a patient from Turkey to be validated by electron microscopy. Furthermore, this case is one of the few reported thyroid angiosarcoma cases in a non-Alpine region.

## Introduction

Angiosarcomas are rare soft tissue neoplasms with an aggressive and disruptive biological behavior, constituting <1% of all sarcomas, worldwide. The majority of angiosarcomas have a skin or soft tissue origin and are generally localized to the head and neck region, or on the lips of patients with lymphedema (1). Angiosarcoma of the thyroid is a rare pathological finding, which has been under debate for ~100 years (2–4). Several studies have reported tumors that exhibit both an anaplastic carcinoma and pseudoangiosarcomatous appearance and thus, it is difficult to classify these tumors as either anaplastic carcinomas of the thyroid or conventional angiosarcomas, according to the WHO classifications (2–4).

Out of 1,271 excised thyroid samples that were assessed over a period of six years (between 2008 and 2013) at the Thyroid Section of the Department of Pathology (Bağcılar Training and Research Hospital, Istanbul, Turkey), only one case of angiosarcoma was determined. Together with a literature review, the current study reports a case of angiosarcoma of the thyroid that was determined by light microscopy, and endothelial differentiation, which was identified by immunohistochemistry and electron microscopy. Written informed consent was obtained from the patient.

## Case report

The patient presented in the current study had previously undergone surgery at a secondary care health center (Başakşehir State Hospital, Istanbul, Turkey) following a diagnosis of undifferentiated thyroid carcinoma. In December 2013, the patient was subsequently referred to the tertiary care center at Bağcılar Training and Research Hospital (Istanbul, Turkey). to receive therapy and consultation. The 62-year-old Turkish female patient had a history of goiter for ~10 years, however, was not on any medication at the time of admission. A swelling had been observed on the right side of the neck, which had grown over the previous few months, however, as the swelling had not compressed the esophagus or trachea, no sign of shortness of breath, difficulty in swallowing or pain was exhibited. When performing laboratory assessments, the thyroid function tests, blood count and coagulation profile were within the normal limits. Laboratory assessments revealed normal levels of free triiodothyronine (2.38 pq/ml; normal range, 2–4.4 pq/ml), free thyroxine (1.16 ng/dl; normal range, 0.9–1.7 ng/dl) and thyroglobulin (7.25 ng/ml; normal range, 3–40 ng/ml). However, a low level of thyroid stimulating hormone was identified (0.01 mU/l; normal range, 0.35–5.5 mU/l). Furthermore, the patient’s hemoglobin level was 13 gm/dl (normal range, 12–16 gm/dl), international normalised ratio was 0.65 (normal range, 0.53–1.62) and activated partial thromboplastin time level was 38.5 sec (normal range, 31.3–54.5 sec). The fine needle aspiration biopsy (FNAB) was rich in blood elements, however, was not diagnostic. The patient did not respond well to the repetition of the aspiration, therefore, elective surgery was proposed and a bilateral thyroidectomy was performed.

**Figure 1:**
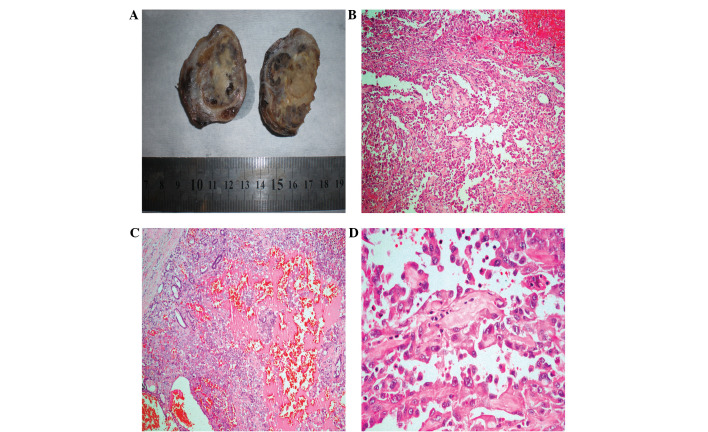
(A) Macroscopic image of thyroid angiosarcoma showing a well-encapsulated nodule that appeared to be cystic and hemorrhagic on the cut surface. Histologically, hematoxylin and eosin staining revealed a (B) peripheral rim of normal thyroid tissue and a central area of hemorrhaging (magnification, ×100) and (C) anastomosing angiomatous structures lined by the neoplastic endothelium (magnification, ×200). (D) High-power view demonstrated large atypical epithelioid tumor cells with prominent nucleoli (magnification, ×400).

Macroscopically, the right lobe (size, 5×5×3.5 cm; weight, 50 g) contained a hemorrhagic nodule measuring 3×2 cm in diameter on the cross section. The left lobe appeared to be normal, weighing 40 g and measuring 4×3×2.5 cm. Paraffin-embedded tissue blocks from the samples obtained by FNAB and multiple samples reprepared from the macroscopic specimen were evaluated together. Microscopically, nodular structures containing intact follicles with fibrous capsules were observed at a number of sites, and exhibited wide hemorrhagic foci at the center. Certain vascular channels formed anastomoses within the bleeding sites, several of which resulted in endothelial proliferation, while other channels were lined by a single layer of epithelial cells. The cells exhibited large vesicular nuclei and prominent macronucleoli (Fig. 1). No capsular invasion or extrathyroidal spread was observed.

**Figure 2:**
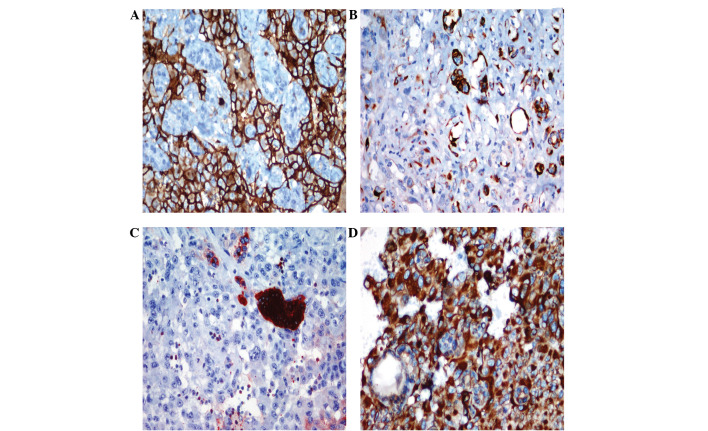
Immunohistochemical staining showed (A) strong diffuse immunoreactivity with CD31 in the membranes of the malignant cells of vascular neoplastic channels, (B) focal strong pan-cytokeratin AE1/AE3 staining, (C) positive staining with thyroglobulin in the abortive follicles and (D) intense staining with vimentin (magnification, ×400).

**Figure 3:**
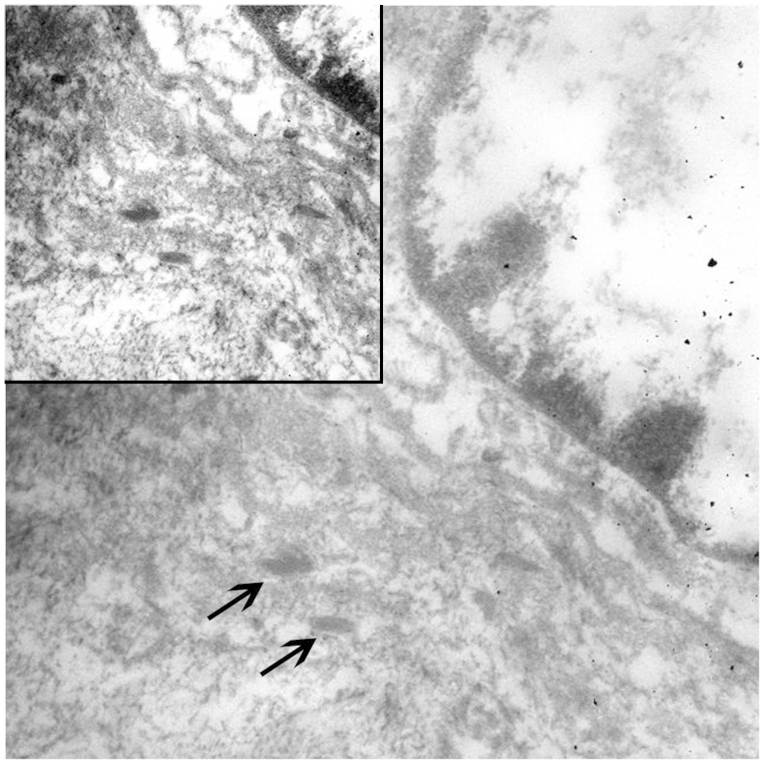
Electron microscopy revealed oval-shaped tumor cells surrounded by continuous basal lamina. Weibel-Palade bodies (arrows) were identified in the cytoplasm of the endothelial cells (magnification, ×60,000; inset, ×40,000).

Immunohistochemistry revealed that the typical cells exhibited strong immunoreactivity for CD31 and vimentin, but weak immunoreactivity for CD34 and factor VIII (FVIII). Strong cytokeratin (CK) AE1/AE3 staining was occasionally observed, as was positive staining for thyroglobulin in the abortive follicles among the atypical cells (Fig. 2). Cells were identified to be negative for TTF-1, which was applied to exclude coexistent follicular carcinoma, and HMB-45, which was applied to exclude malignant melanoma. A diagnosis of angiosarcoma was determined based on these characteristics and upon observation of Weibel-Palade bodies, which exhibited endothelial differentiation that was observed under an electron microscope (Fig. 3).

**Figure 4:**
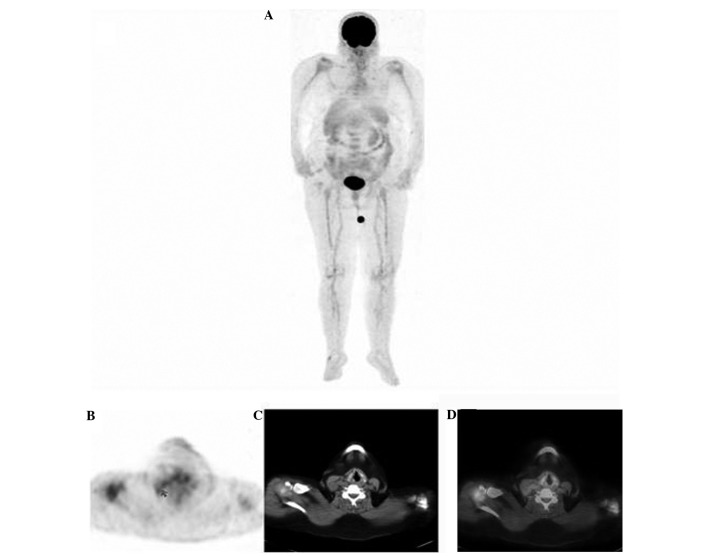
Postoperative positron emission tomography (PET)/computed tomography (CT) maximum intensity projection images. (A) A hypodense lesion showed peripheral minimal hypermetabolism in the right inferior jugular area, compatible with postopreative changes or tumor residue. (B) PET image revealing minimal hypermetabolism with a low fluorodeoxyglucose uptake in same area, (C) CT image and (D) fusion images of the axial cross section revealing a hypodense lesion in the area of the right inferior jugular.

At 40 days following the surgery and staging assessments, whole body imaging with positron emission tomography-computed tomography was conducted 1 h following the injection of 11.66 mCi F-18 fluorodeoxyglucose (FDG), while the fasting blood glucose level was 113 mg/dl. A hypodense lesion, 20×22 mm in size, in the right inferior jugular area was detected, revealing a peripheral hypermetabolism with a low intensity signal, compatible with the collection area. Furthermore, lymphadenopathies were identified in the bilateral jugular chain, which did not exhibit pathological FDG involvement (Fig. 4). The patient refused additional complementary surgery and chemotherapy and at the 15-month follow-up reported no health issues regarding the angiosarcoma.

## Discussion

Angiosarcoma of the thyroid, a rare type of sarcoma, was originally reported in a patient from the mountainous Alpine region 90 years ago (3,5). Based on a search conducted using PubMed, to date 48 cases have been reported in the literature (6). A limited number of cases from non-Alpine regions have previously been reported (5,7), however, to the best of our knowledge, this is the first reported case of angiosarcoma of the thyroid in Turkey from a non-Alpine region, which was identified by electron microscopy.

In total, 2–10% of all malignant thyroid tumors are observed with a high incidence in Alpine regions, including Switzerland, North Italy and Austria (2,5,7). The patient in the current study had a 10-year history of goiter and was from the Black Sea region, an endemic goiter region of Turkey. Although iodine deficiency may be a factor in the etiology of angiosarcoma, the identification of cases reported from a non-Alpine region leads to the consideration that additional factors may also have an impact.

The female gender had a marked predominance in the distribution of cases presented in the literature and the mean age in the Alpine regions was 60 years, whereas the mean age was 65.5 years in the non-Alpine regions (7,8). In a recent study and literature review, it was reported that angiosarcomas of the thyroid were most frequently identified in females aged >60 years (6). Thus, the patient in the present study was comparable with those from the endemic regions in Europe, with regard to age and gender.

The diagnosis of angiosarcoma is challenging for clinicians and pathologists and is one of the most debated vascular pathologies of the thyroid, when observed in patients from unusual regions. Certain authors question the existence of angiosarcomas (4); while others hypothesize that the reported cases were neoplasms exhibiting angiomatoid characteristics of anaplastic or undifferentiated carcinomas (2–4). Additionally, previous studies have reported that the immunohistochemical detection of endothelial differentiation and the observation of Weibel-Palade bodies, via electron microscopy, supports the endothelial origins of epithelioid angiosarcomas (5,9,10). In the present case, the diagnosis was determined by electron microscopic assessment.

When vascular pathologies are exhibited in the thyroid, immunohistochemical markers that are frequently used by pathologists may facilitate the differential diagnosis and determination of a diagnosis of angiosarcoma. The highly sensitive CD31 antibody is expressed in 90% of angiosarcomas, whereas it is expressed in ~1% of carcinomas (1). The immunohistochemical detection of FVIII-related antigens and the ultrastructural identification of Weibel-Palade bodies confirmed the occurence of endothelial differention in neoplastic cells (2,9). Therefore, the endothelial markers, CD31 and FVIII, in addition to immunopositivity for vascular markers, CD34 and FVIII related antigen, may be added to the immunohistochemical panel when determining a diagnosis of angiosarcoma (11,12). However, the staining may be associated with marked platelet uptake (2), which may cause confusion during the diagnosis of angiosarcomas. Additionally, cases that are positive for pan-CK are determined to be epithelioid angiosarcoma and should also be taken into consideration (10); strong pan-CK staining was locally observed in the present case.

It is hypothesized that angiosarcomas are transitional tumors, which exhibit variable presentations of mesenchymal metaplasia with endothelial and epithelial differentiation. Angiosarcomas have not yet been included in the WHO classification of thyroid tumors (2004) and have instead been classified under other rare thyroid malignancies of the four major groups (papillary, follicular, medullar and anaplastic carcinomas) (13). Despite the continuation of nosological issues and regardless of where this sarcoma is grouped, it appears that the distinction of this type of tumor, which possesses similar prognosis and treatment options to other types of tumor, remains a topic for academic debate. Although the common treatment approach is multimodal therapy, which involves obtaining negative surgical margins and administering adjuvant chemotherapy with or without radiotherapy, the survival rates are limited to just a few months (5,7).

In the current case, surgery was performed at an external center; however, no extrathyroidal spreading was exhibited and a clean surgical margin was achieved. The patient refused the additional complementary surgery and chemotherapy options, and is currently disease-free.

In conclusion, angiosarcoma of the thyroid is a type of head and neck neoplasm with a poor prognosis, which morphologically resembles a soft tissue sarcoma, and exhibits epithelial and endothelial differentiation, as well as immunoreactivity for pan-CK. In addition, CD31 immunohistochemistry was identified to be valuable in the differential diagnosis; however, a variety of markers are required for the diagnosis of angiosarcoma, such as CD34, FVIII, vimentin and pan-CK. Furthermore, it was established that electron microscopic assessment may assist with endothelial differentiation. Finally, the possibility of angiosarcoma presenting in patients with a long history of goiter and who originate from a region of endemic goiter, must be considered.
